# My continuing adventure with 21 CFR Part 11 - the evolution of Zymark's compliance

**DOI:** 10.1155/S1463924601000232

**Published:** 2001

**Authors:** Stephen M. Dobro

**Affiliations:** Zymark Corporation Hopkinton MA 01748 USA

## Abstract

A renewed focus has been given to the 3-year-old regulation 21 CFR Part 11, Electronic Records and Electronic Signatures. This paper gives a chronology of the process of an equipment vendor, Zymark Corporation, validating laboratory automation equipment for compliance to the regulation 21 CFR Part 11. Zymark's Tablet Processing Workstation II™ (TPW™) and Prelude™ are the instruments chronicled. The first instrument, the TPW™, was developed before Zymark defined its strategy on how to meet its customer's need for 21 CFR Part 11 compliant equipment. The TPW™ has been available for several years, and in the summer of 1999 it received a major software upgrade to improve its security. The second instrument, the Prelude™, is a new product. It had a design requirement to meet the regulation. Zymark's Part 11 strategy was already in place and used for this development project. This chronology will include all aspects of the exercise, including familiarization with the standard, development of the protocols, review of the protocols by industry experts, review of the protocols by pharmaceutical users, execution of the tests, preparation of the exception reports, and the release of any necessary product revisions.


Journal of Automated Methods & Management in Chemistry, Vol. 23, No. 6 (November–December 2001) pp. 183–187

### My continuing adventure with 21 CFR Part 11—the evolution of Zymark's compliance†

**Stephen M. Dobro**

*Zymark Corporation, Hopkinton, MA 01748, USA. e-mail: stephen.dobro@zymark.com*

*A renewed focus has been given to the 3-year-old regulation 21 CFR Part 11, Electronic Records and Electronic Signatures. This paper gives a chronology of the process of an equipment vendor, Zymark Corporation, validating laboratory automation equipment for compliance to the regulation 21 CFR Part 11. Zymark's Tablet Processing Workstation II™ (TPW™) and Prelude™ are the instruments chronicled. The first instrument, the TPW™, was developed before Zymark defined its strategy on how to meet its customer's need for 21 CFR Part 11 compliant equipment. The TPW™ has been available for several years, and in the summer of 1999 it received a major software upgrade to improve its security. The second instrument, the Prelude™, is a new product. It had a design requirement to meet the regulation. Zymark's Part 11 strategy was already in place and used for this development project. This chronology will include all aspects of the exercise, including familiarization with the standard, development of the protocols, review of the protocols by industry experts, review of the protocols by pharmaceutical users, execution of the tests, preparation of the exception reports, and the release of any necessary product revisions.*

### Introduction

This is the story of the process that Zymark's Manager of Product Testing and Validation, Stephen M. Dobro, used to research regulation 21 CFR Part 11 and evaluate two of Zymark's products for compliance with it. The exercise created the process that Zymark now uses to ensure compliance for all its products whose use fall under the requirements of 21 CFR Part 11. Throughout this document, the term 'the regulation' and 'Part 11' will be used interchangeably with 21 CFR Part 11.

The information is presented in chronological order. The major topics include the following.

- Motivation and goals: why Zymark got involved with this project and what it intended to accomplish.
- Understanding the regulation: what knowledge was necessary to complete the project successfully.
- Developing the validation plan: the author's experience developing a protocol—how he went about it and what questions came up.
- Expert and customer review: the author indicates what others thought about his drafts of the protocol and how those comments shaped the plan.
- Execution and results: the author shows how the actual testing went.

† This paper was initially presented at the ISLAR 2000 Conference and is reproduced here by kind permission of Zymark Corporation.

### Motivation and goals

What is Zymark's motivation for compliance with the regulation? Zymark provides automation equipment for use in research and analytical laboratories. Its customers need to comply with the regulation and it is in Zymark's best interest to do all that it can to meet customers' needs. In recent years, Zymark has been changing from an engineering- to a market-driven company. Engineering-driven means that the engineering department comes up with new product ideas. Market-driven means that one listens to customers for new product ideas as well as ideas for quality improvements. The quality department has been making changes to help Zymark adapt to this new philosophy. To be a successful market-driven company, one needs to open up and learn to listen to customers. During 1999, the quality department spent a lot of time talking with customers to find out where to target improvements.

Software issues were one of the major themes reflected in these customer surveys. Upon joining the quality department in January 1999, the author's first assignment was to evaluate Zymark's process for software quality assurance. Software is the author's specialty, having spent more than 10 years as a software developer. Software quality was a topic that was coming up quite frequently with customers. Not only was software quality an issue, the author heard about 21 CFR Part 11 from every customer who either came in for an audit or sent an audit questionnaire to Zymark. The bottom line was that Zymark's customers needed to comply with the regulation. The project was added to the author's list of projects.

Very specific goals were set up for the project. The first goal was to create a standard validation plan that could be used to evaluate existing products as well as new products. It had to be generic enough so that it could easily be adapted for any Zymark product. A second goal was to create a resulting package that would be of value to Zymark's customers. It would not be good enough just to meet the letter of the regulation for it was important to satisfy customers' needs on this topic. After all, it is customers that have to answer to the FDA. Zymark wanted to improve their products and wanted their customers to believe that the testing done was good testing. The goal was to make it good enough so that Zymark's customers would like to have their own copy to reference in their documentation.

http://www.tandf.co.uk/journals
DOI: 10.1080/14639240110092558

### Understanding the regulation

Probably the largest task associated with the project was to understand the regulation. There were several information sources used, which included government regulations, FDA guidance documents, magazine articles, individual experience, and, most importantly, the author's previous work experience was in non-regulated industries, so he was about to get a real learning experience. The regulation, which he printed from the FDA web page, was a thick document, but the regulation itself was only the last few pages. The task, however, was still a lengthy process.

From there, the author reviewed guidance documents, magazine articles, information from industry consultants, and anything else on the subject. He also reviewed information that had been given to Zymark from customers. Some customers had already put together their own set of guidelines, and these were beneficial. Being new to the industry, the author also needed to learn about computer system validation and how it related to 21 CFR Part 211, Current Good Manufacturing Practice for Finished Pharmaceuticals. He spoke to people at Zymark who were familiar with pharmaceutical validations, including the field service engineers who perform Installation Qualification and Operational Qualification when Zymark equipment is installed. Zymark also has chemists who perform methods transfers from manual execution to automated equipment. This education was essential to understanding how Part 11 fitted into the scheme of these regulations.

As the author was gathering information, he started to make a list of questions. One category of questions involved finding different interpretations of the regulation from the various information sources. An example of this is the level of security necessary: how secure does the system have to be? Data files on a personal computer are difficult to protect. Zymark's design put the audit log and data records in a SQL Server database, which provides for tight security. But how secure do other system files need to be? Some questions did not have simple, straightforward answers. A second example involved unattended terminals. If someone logs into a system and then leaves the terminal unattended, what should happen if a different operator steps up to the terminal? The author later came across an FDA guideline that helped him make a decision on this point. As it turns out, solutions can be implemented in many different ways. A third example is system configuration. For Zymark products, the administrator can configure what operations will be password-protected. This means that the system can be configured to have very tight or minimal security. On this topic, one of Zymark's customers stated that if it was possible for the system to be configured not to comply with the regulation, then it is a non-compliant system. These differences in interpretation were part of the struggle in coming to terms with the regulation. A second category of questions centred on terminology. There were many phrases in the regulation that were unclear. Some examples are: 'What is meant by an "accurate copy" of records?' and 'What is a "device check"?' Gathering information was more than just a project phase; it was a continuous process of learning. For the author, that was about 18 months of education so far!

### Developing the validation plan

Although the author still had many unanswered questions, he began to develop the validation plan. This was a two-step process. The first step was to create a validation plan that listed generic test cases that could be used for any product. The author made this list of test cases generic even though he knew which product he would be testing first. He wanted to be sure he came up with a list of test cases that could be used for any product. When he finished the generic validation plan, he had a template that could be used for step two. The second step was to use this template to create a specific validation plan for the actual product to be tested. Each step is described in more detail below.

When creating the generic validation plan, the author had to answer some questions on the scope of the plan. It was decided the generic plan should cover all Zymark products. Next, did the plan need to address electronic records, electronic signatures or both? This could vary depending on the product. At first, the author thought that only the electronic records portion would apply to Zymark products. As he learned more, it became clear that electronic signatures also needed to be addressed. Zymark used different levels of security. Those with proper access could change parameters, run methods or administer the system. The next question that had to be answered was whether the scope should include open systems or just closed systems. All current products and plans were for products that were closed systems. After answering these questions, it was time to define generic test cases that would address all of the specific product requirements related to the regulation.

Before the author defined the test cases, it was necessary to create a requirements traceability matrix. A requirements traceability matrix is a table where the first column lists all of the requirements and the second column lists one or more test cases for each requirement. Using this matrix, one can be sure they provide at least one test for each requirement. First, the author listed in the table all the requirements from the regulation for closed systems and for signature security. He took these verbatim from the regulation. Then he carefully thought out generic tests that would prove meeting the requirement and listed these in the second column. For example, 11.10c reads 'Protection of records to enable their accurate and ready retrieval throughout the records retention period'. Dobro defined two generic tests: (1) new data records must not overwrite old data records and (2) unauthorized users must not be able to modify or remove data records. When the matrix was complete, so was the generic test plan. It was now time to adapt the plan for the specific product.

The first product to test was the Tablet Processing Workstation II™ (TPW™). The TPW™ was chosen first mainly for two reasons. First, it was an existing or legacy product developed before the existence of the regulation whose primary focus was for use in Pharma-

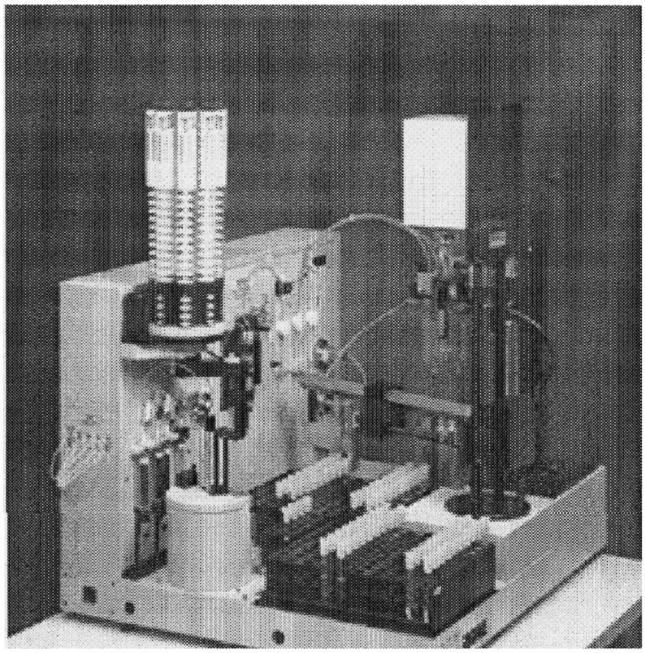

*Figure 1. TPW™ Robotic Unit.*

ceutical QC labs. Customers had been asking for our plans to make it compliant. Second, it had been given a recent software upgrade that incorporated a brand-new security module.

Months later, the author adapted the plan to apply to the Prelude™, a brand-new product. This was the first product that listed the regulation as a product requirement. Details of the Prelude™ case follow the TPW™ case.

To document and test a product properly, it helps to understand its functionality and data architecture. The primary applications of the TPW™ are sample preparation for assay, content uniformity, and stability testing. The Unit performs basic functions such as table dispersing, dilution and filtration of samples. It can then feed the sample into a measuring device such as an HPLC or UV, or it can store the sample using a sample collection product called EasyFill™.

Figure 1 shows the TPW™ Robotic Unit. The hardware includes test tube racks, robotic arm, filter dispenser, disperser station, which contains a three-place balance, and a dispense station, which contains a four-place balance.

To give an understanding of how the TPW™ handles data, see figure 2. There are three boxes in the data architecture diagram: the PC, the Robotic Unit and the output device. The blocks within the PC show data. Methods and solvent density are saved in individual files. Data records are produced and stored locally in a SQL Server™ Database and Excel spreadsheet. Copies of the data can also be sent to another database or spreadsheet using the Advanced Data Channel option that will be discussed later. Audit trail information is stored locally in the SQL Server™ Database.

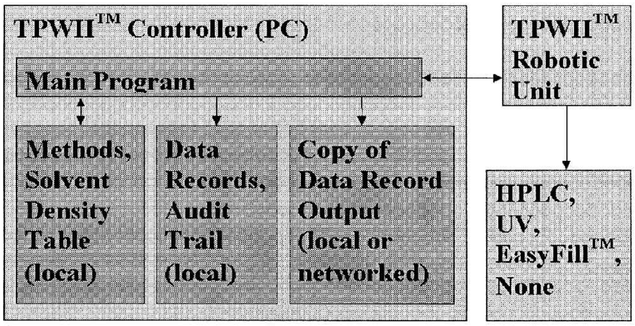

*Figure 2. TPW™ data architecture.*

With general product knowledge, the specific test cases for a product can be put together. The author was faced with more questions. 'Can the plan just reference design level testing?' When the TPW™ was developed, a lot of testing, called design-level testing, was performed. The author had this idea that he could just reference those test results in this plan. It did not take long to realize that this was not a good idea. It would make it difficult to audit the testing and to reuse the results. Also, the testing done for legacy products like the TPW™ was bound to be incomplete. It was decided that this test plan should stand on its own.

'Does this test plan need to cover every type of operation that the system can perform?' For the TPW™, just about every operation caused data to be recorded. Instead of making sure that every operation was tested (a duplication of the design level testing done at the time of development), it was decided that the testing would include a typical set of operations.

'What are the instrument boundaries?' During the creation of the generic test cases, a line had to be drawn between the TPW™ and external equipment—this was defined as the instrument boundary. Two examples are now discussed.

The first example is advanced data channels, which is an available option for the system. Advanced data channels allow the user to store data outside of the TPW™'s secure database—perhaps even on a system network. It was decided that this is only a copy of the secure record and, therefore, is not within the instrument boundary. The second example is data from detection devices such as an HPLC or UV. Were the data from these devices part of the system? The answer to this was no, since the data from these devices were not returned to the TPW™. Once these questions were answered, the author then adapted the generic test cases to the TPW™. This was a relatively short process compared with what was needed to understand the regulation.

### Expert and customer review

Once a good draft of the validation plan was together, which included detailed step-by-step procedures for each test case, the next step was to have it reviewed by an outside expert. The author utilized a well-known pharmaceutical industry consulting group. To help answer questions, the consultants told the author to keep in mind the purpose of the regulation. The purpose can be summarized as follows: (1) make sure that electronic records and signatures are as credible as paper records and signatures and (2) make sure the FDA can access the information. This turned out to be good advice, because the author found that it was much easier to interpret the details when he kept that overall purpose in mind.

The experts also gave two pieces of advice for the test plan. This first was to give an overview of the product. This should include what the instrument does and how data are stored and manipulated. The idea is that when someone reads the document for the first time, they can then easily relate between the regulation and the tests performed on the product. The second suggestion was that the test cases be organized in a more logical manner. Originally the test cases were ordered to match that of the sections from the requirement. This turned out to be haphazard. The author changed the order to represent how the user would approach the machine. First, they would login, so the first tests were for system security. Next, they would configure the system, so the next tests were for proper security-level checking. The changes were made. It was now time for a review by Zymark's customers.

Zymark's customers were very happy to give feedback on the test plan. They were thrilled that Zymark was willing to ask for it! One customer gave a really detailed critique of the test cases. The customer let the author know that he should not assume that the system would work a certain way—even if it were a function of the PC's operating system. The customer said to make sure the test was only against defined requirements. Another suggestion was to record the test results using screen captures. This was actually done for the TPW™ V2.0 testing. From other suggestions, it was clear that they wanted the tests to be very detailed. This was a direct reflection on what customers felt the FDA would like to see.

### Execution and results

Finally, it was time to run the validation. As is always the case with a validation or testing project, preparation is most of the work! The testing turned out to be very useful. One non-compliance issue was discovered—some of the failed login attempts were not recorded to the audit trail. It would record only the last failed attempt in a series of tries. The testing also exposed some suggested improvements. A suggested improvement is something not required by the regulation but which is functionality suggested by customers. Zymark's goal was to satisfy its customers, so these suggestions were taken very seriously. Another positive outcome of the exercise was good feedback on usability. By performing the testing, the author discovered where improvements could be made on the usability of the product.

The results showed that the product was not perfect. Zymark used this information to create and release a software patch that made the product 100% 21 CFR Part 11 compliant.

### Leveraging results from the project

A few months later, the author had the chance to test another product for Part 11 compliance. This was for the new product called Prelude™. Prelude™ is the next generation of Zymark's BenchMate™ product. It is a personal automation tool for the chemist. The question was 'Can the validation work from the TPWII™ be leveraged for the Prelude™?' A lot of time was put into creating the generic plan for testing Part 11 compliance. It was interesting to see if it paid off for the second product to be tested.

First review the Prelude™ functionality and data architecture. The primary applications of the Prelude™ included a wide range of dilutions, standard preparation, drug substance analysis, potency assays and solubility testing. It can feed samples into a measuring device such as an HPLC or UV, or it can store the sample using a sample collection product call the EasyFill™.

Figure 3 shows the Prelude™ Robotic Unit. The hardware includes test tube racks, robotic arm, filter dispenser, a dispense station which includes a four or five-place balance, and a barcode reader. Options that will become available soon include a sonication station. The Architecture is identical to the TPW™ (figure 4). That made creating the test procedures easy!

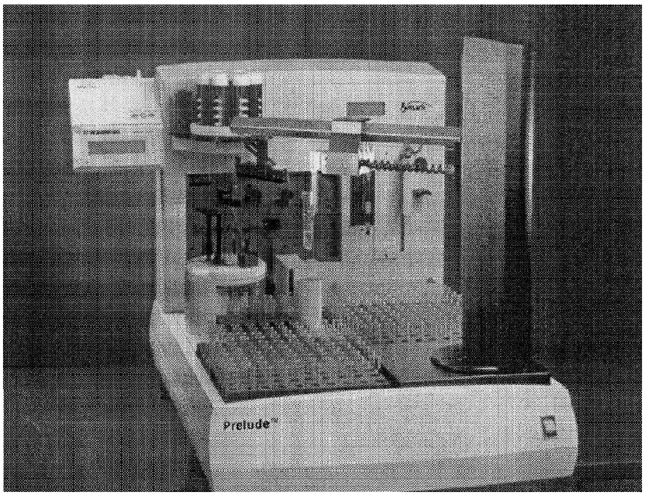

*Figure 3. Prelude™ Robotic Unit.*

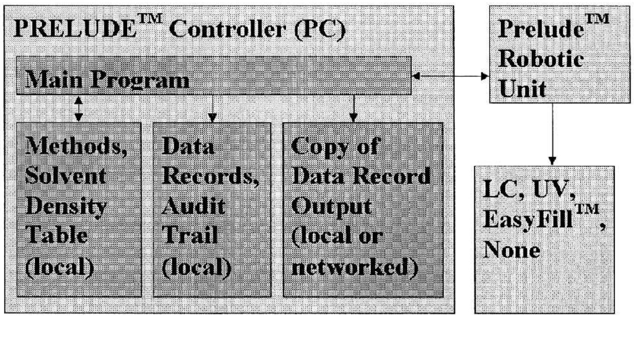

*Figure 4. Prelude™ data architecture.*

The results for the Prelude™ were perfect. It was 100% compliant from the first day of its life. By having a test standard in place for Part 11 compliance, the product was designed to meet the regulation. In this case, since the product architecture was identical to the TPW™, the test plan, procedures and results were designed, documented and executed in less than a week. To give more data behind the leveraging of the generic test plan, the author is currently working on a test plan for an upgrade to Zymark's MultiDose® product. This product has a different architecture than the TPW™ or the Prelude™. It is estimated that the design of the test plan/procedure will take about 3 weeks.

### Summary

Zymark is now positioned to assure compliance for all its products—whether it be upgrading existing products or creating new ones. The plan that was put together is a roadmap that will be used. Zymark learned that the regulation can be addressed with an isolated workstation—it was addressed successfully with the TPW™ and Prelude™. The value of customers input was incredible. It is vital really to understand customers' needs to meet those needs. Finally, this exercise helped Zymark improve the partnership it has with its customers. Zymark's customers define the quality of Zymark's products. Zymark is now ready to meet their customers' needs better.

